# Radiological Outcomes and Approach-Related Complications in Oblique Lateral Interbody Fusion at the Upper Lumbar Level

**DOI:** 10.3390/jcm14103333

**Published:** 2025-05-10

**Authors:** Hee-Woong Chung, Han-Dong Lee, Myungsub Lee, Nam-Su Chung

**Affiliations:** Department of Orthopaedic Surgery, Ajou University School of Medicine, 164 World Cup-ro, Suwon 16499, Republic of Korea; gmldnd0324@naver.com (H.-W.C.); handonglee@gmail.com (H.-D.L.); su720126@hanmail.net (M.L.)

**Keywords:** oblique lateral interbody fusion, upper lumbar spine, radiologic outcome, approach-related complication

## Abstract

**Background/Objectives:** Despite recent advances in minimally invasive extrapleural lateral approaches, oblique lateral interbody fusion (OLIF) at the upper lumbar level is often difficult and limited to optimal reconstruction. We aimed to compare the radiological outcomes and approach-related complications of OLIF between the upper (L1–2 or L2–3) and lower (L3–4 or L4–5) levels. **Methods:** This study is a retrospective review of OLIF in the upper (*n* = 63) and lower (*n* = 60) lumbar level groups. Radiological parameters included the anterior/posterior disc height, coronal/sagittal disc angle, cage position, cage subsidence, and fusion rate at a postoperative 1-year follow-up. Approach-related complications including pleural/peritoneal lacerations, neurovascular injury, and other organ injuries were examined. **Results:** The baseline radiological parameters were similar between the two groups (all *p* > 0.05). At 1-year postoperatively, the anterior disc height (ADH) was significantly greater in the lower-level group (*p* = 0.031), while no significant differences were observed in the posterior disc height, coronal/sagittal disc angle, cage anterior position, or cage subsidence rate (all *p* > 0.05). The fusion rates were 97.9% and 95.0% at the upper and lower lumbar levels, respectively (*p* = 0.146). During OLIF at the upper lumbar level, chest tube insertion due to pleural laceration was observed in 11 (17.5%) cases. One case (1.2%) of segmental artery injury and two cases (3.2%) of pseudo-hernia were attributed to iliohypogastric nerve injury. **Conclusions:** Although the extrapleural approach in OLIF at the upper lumbar level is often limited, the radiological outcomes were comparable to those of OLIF at the lower lumbar level.

## 1. Introduction

Oblique lateral interbody fusion (OLIF) is a relatively recent minimally invasive interbody fusion technique that has been widely used for treating lumbar spinal conditions, such as degenerative disc disease, adjacent segment disease, and spinal deformity. By employing an oblique surgical trajectory through the retroperitoneal space, OLIF minimizes direct manipulation of the psoas muscle and neural structures. This approach provides several well-recognized advantages, such as reduced operative times, lower intraoperative blood loss, and effective indirect neural decompression, achieved through the restoration of the intervertebral disc height [1,2,3]. While the safety and efficacy of OLIF has been well established at the lower lumbar level (L3–4 or L4–5), its application in the upper lumbar level (L1–2 or L2–3) remains challenging, owing to anatomical constraints and surgical access limitations, including the presence of the rib cage, pleura, and diaphragm.

Despite recent significant advancements in the extrapleural lateral approach, most studies have focused on trauma cases [4,5], while performing OLIF in the upper lumbar spine presents unique challenges compared with trauma surgeries, as it requires a small incision and placement of a tubular retractor to establish an adequate working space. If the tubular retractor is placed improperly, complications such as endplate injury and cage malpositioning may occur [6]. Although fusion of the upper lumbar spine can be performed using posterolateral fusion or posterior lumbar interbody fusion, OLIF may be a more favorable option, particularly for multilevel reconstruction, as it offers lower surgical invasiveness and superior effectiveness in restoring sagittal alignment, including lordosis correction [7,8]. Despite these theoretical advantages, published evidence regarding OLIF in the upper lumbar spine remains scarce. Most recent studies have focused narrowly on the technical feasibility of the surgical approach itself, without a comprehensive evaluation of clinical and radiological outcomes or procedure-specific complications [9,10].

Therefore, the present study aims to bridge this gap in the literature by comparing the radiological outcomes and approach-related complications of OLIF performed at upper lumbar levels with those observed at lower lumbar levels. Through this comparative analysis, we seek to evaluate the safety, feasibility, and clinical utility of OLIF as a viable surgical option for interbody fusion in the upper lumbar spine.

## 2. Materials and Methods

### 2.1. Patients

Institutional review board approval was obtained before conducting the study (AJOUIRB-DB-2025-075). This retrospective study included 123 consecutive patients (46 males and 77 females) who underwent one- or two-level OLIF procedures at either the upper lumbar spine (L1–2, *n* = 15; L2–3, *n* = 48) or the lower lumbar spine (L3–4, *n* = 30; L4–5, *n* = 30) at a single institution between December 2014 and December 2023. All patients had a minimum follow-up duration of 1 year after the index surgery. The mean age at surgery and mean follow-up period were 65.9 ± 10.4 years (range: 46 to 86) and 61.1 ± 18.3 months (range: 12 to 112), respectively. All patients were diagnosed with lumbar degenerative disease, including spondylolisthesis, spinal stenosis, and degenerative disc disease, accompanied by severe pain. They had undergone conservative treatments such as medication, physical therapy, and epidural injections for at least 3 months but showed inadequate response. Patients with incomplete data or a history of prior lumbar spinal surgery were excluded. Additionally, those who had undergone surgery for spinal tumors, vertebral fractures, or spinal infections were also excluded from the study. Routine preoperative imaging included standing dynamic lumbar spine radiographs (anteroposterior [AP], lateral, flexion, extension views), whole-spine AP/lateral radiographs, computed tomography (CT), and magnetic resonance imaging. Immediately after surgery, lumbar AP and lateral radiographs, as well as CT scans, were routinely obtained to confirm cage placement and detect any complications. All patients wore a thoracolumbosacral orthosis for 3 months postoperatively and underwent regular outpatient follow-up evaluations at 1, 3, 6, and 12 months. A follow-up CT was performed between the six-month and one-year follow-up visits. Demographic data, including patients’ body mass index (BMI), bone mineral density (BMD), smoking history, preoperative diagnosis, operative time, estimated blood loss (EBL), and approach-related complications were examined. For cases involving two-level OLIF procedures, the operative time and EBL were adjusted per level for statistical analysis.

### 2.2. Surgical Technique

**Figure 1:**
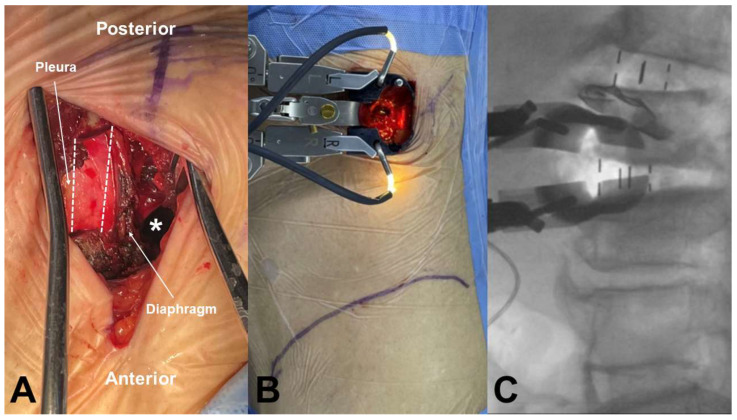
OLIF approach at upper lumbar spine. (**A**) The retroperitoneal space was accessed by resecting the 11th rib (dotted line) without pleura laceration and retracting the diaphragm anteriorly (white asterisk; retroperitoneal space). (**B**) The tubular retractor was mounted at the L1–2 disc space. (**C**) Intraoperative fluoroscopic images of OLIF at L2–3, performed through the same incision after completing OLIF at L1–2.

All surgical procedures were conducted by a single experienced spine surgeon (N.C.) following the original OLIF technique described by Hynes [1]. Under general anesthesia, patients were positioned in the right lateral decubitus posture at 90 degrees on a Jackson Spinal Surgery Table (Mizuho OSI, Union City, CA, USA). The exact lateral position was verified using AP and lateral fluoroscopic guidance with a C-arm. In the upper lumbar spine, the L1–2 (or L2–3) disc level was identified and marked under the C-arm. After palpating the ribs around the target level, a skin incision approximately 4 cm in length was made along the direction of the rib. If the surgical trajectory was obstructed by the rib cage, we performed partial resection of the 11th or 12th rib. Following this, the diaphragm was carefully retracted anteriorly to access the retroperitoneal space. The target disc was then exposed using the OLIF25 tubular retractor system (Medtronic Inc., Minneapolis, MN, USA) via a retroperitoneal antepsoas approach (Figure 1A,B).

Extensive discectomy and removal of the cartilaginous endplates were performed to prepare for OLIF cage insertion, with meticulous care taken to preserve the bony endplates. Contralateral annular release was performed using a blunt-tipped shaver with gentle hand tapping, followed by sequential trial insertions to distract the disc space. A lateral polyetheretherketone cage (Clydesdale; Medtronic Inc.) filled with autologous iliac bone and a demineralized bone matrix (Grafton; Medtronic Inc.) was inserted obliquely into the disc space and rotated orthogonally in the true lateral direction (Figure 1C). Under AP C-arm fluoroscopic guidance, the cage was advanced to the midline of the disc space in a press-fit manner. If a pleural laceration occurred during the anterior approach, a chest tube was placed after cage insertion. Following this, primary repair of the pleura was performed, and the muscles were closed layer by layer. Upon completion of the anterior procedure, the patient was repositioned into the prone position, and supplemental posterior pedicle screw fixation was performed using either an open or a percutaneous technique. In cases of severe central canal stenosis [11], in which the dural sac showed a homogeneous gray signal with no visible CSF or rootlets, additional laminectomy was carried out.

For the lower lumbar spine, the target disc level (L3–4 or L4–5) was first identified and marked, followed by a longitudinal skin incision made approximately two-finger widths anterior to the anterior margin of the disc space. All subsequent procedures were performed in the same manner as for the upper lumbar spine, except for rib resection.

### 2.3. Radiographical Measurement

Anterior and posterior disc heights (ADH and PDH), as well as coronal and sagittal disc angles, were measured preoperatively and postoperatively using whole-spine standing X-rays. The cage anterior position was determined by measuring the distance from the anterior border of the inferior vertebral body on midsagittal postoperative CT reconstructions, as previously described [12]. The fusion status was assessed based on the classification system described by Bridwell et al. [13], with grades 1 and 2 regarded as indicative of successful interbody fusion. Cage subsidence was assessed using postoperative and serial follow-up radiographs and was defined as a decrease in height ≥ 2 mm due to the cage sinking into an adjacent vertebral body, as determined by comparison with previous radiographs [14]. Radiological parameters were independently measured by a board-certified spine surgeon (H.C.), who was not involved in the clinical care or surgical treatment of the study participants to minimize observer bias. All measurements were performed using a standardized picture archiving and communication system (INFINITT PACS; INFINITT, Seoul, Republic of Korea).

### 2.4. Statistical Analysis

Descriptive statistics are presented as frequencies (%) for categorical variables and as means ± standard deviations for continuous variables. Comparisons between groups were conducted using the chi-square test for categorical variables and the independent *t*-test for continuous variables. All statistical procedures were carried out using the SPSS software for Windows (version 20.0; IBM Corp., Armonk, NY, USA). Statistical significance was set at *p* value < 0.05.

## 3. Results

### 3.1. Subject Demographics

Table 1 summarizes the baseline demographic characteristics and surgical parameters of the patients included in this study. A total of 123 patients were included, with 63 in the upper-level group and 60 in the lower-level group. There were no statistically significant differences in baseline characteristics, such as age (66.3 ± 11.9 vs. 65.9 ± 9.4 years), sex ratio, BMI, BMD, smoking history, or follow-up duration (all *p* > 0.05). Regarding preoperative diagnoses, spondylolisthesis was the most common indication in both groups, followed by spinal stenosis and degenerative disc disease, with no significant intergroup difference (*p* = 0.486). The proportion of patients undergoing one- versus two-level OLIF procedures was also similar between groups (*p* = 0.122). The operative time and EBL did not differ significantly either (*p* = 0.101 and *p* = 0.451, respectively).

### 3.2. Approach-Related Complications in OLIF at the Upper Lumbar Level

Among the 63 patients who underwent OLIF at the upper lumbar level, rib resection was required in 16 patients (25.4%) during the surgical approach. Specifically, rib resection was performed in 8 cases at the L1–2 level and in 8 cases at the L2–3 level. At L1–2, the 11th rib was resected in 6 cases and the 12th rib in 2 cases, whereas at L2–3, the 11th rib was resected in 5 cases and the 12th rib in 3 cases. Chest tube insertion was required in 11 cases (17.5%) due to pleural laceration. Intraoperatively, pleural lacerations occurred in 3 cases at the L1–2 level (20.0%) and 6 cases at the L2–3 level (12.5%), necessitating immediate chest tube placement. Additionally, in 1 case at each level, a postoperative chest radiograph revealed pneumothorax, requiring chest tube insertion. One case (1.6%) of segmental artery injury had occurred intraoperatively, and 2 cases (3.2%) of pseudo-hernia due to iliohypogastric nerve injury were identified during the outpatient follow-up period. Both patients exhibited symptomatic improvement within 6 months of the application of an abdominal binder (Table 2).

### 3.3. Radiological Outcomes

The radiological outcomes of the two groups are shown in Table 3. There were no significant differences in preoperative ADH, PDH, coronal disc angle, or sagittal disc angle (all *p* > 0.05). Although statistical comparisons were not performed, the postoperative ADH, PDH, and sagittal disc angle increased in both groups compared to the preoperative values, reflecting effective disc height restoration and sagittal alignment improvement achieved through OLIF. At 1 year postoperatively, only ADH was significantly greater in the lower-level group (11.7 ± 2.3 vs. 13.1 ± 3.2; *p* = 0.031). Among the cage parameters, the cage height tended to be higher in the lower-level group (*p* < 0.001). There were no significant differences in the cage anterior position, subsidence, or fusion rate between the two groups (all *p* > 0.05).

## 4. Discussion

Since its introduction by Capener in 1932 [15], anterior lumbar fusion has undergone significant advancements, evolving from minimally invasive anterior lumbar interbody fusion (ALIF) to OLIF in 2012 [16]. Because OLIF utilizes the retroperitoneal antepsoas approach, it has a lower risk of vessel and bowel injury than transperitoneal ALIF, and it reduces the risk of lumbar plexus injury and muscle damage compared with direct lateral interbody fusion [2]. Additionally, compared with posterior surgeries, such as posterior lumbar interbody fusion or transforaminal lumbar interbody fusion, OLIF offers advantages in multilevel surgeries, owing to reduced blood loss, shorter operative times, and greater lordosis restoration [7,17,18].

However, most studies on OLIF have been conducted at the lower lumbar level, and its application in the upper lumbar spine remains limited. This is because the incision site is often obscured by the rib cage, and the presence of the diaphragm makes this approach more challenging. The thoracolumbar junction can be accessed through a transpleural approach. However, this method is also associated with postoperative pulmonary complications, such as atelectasis, pneumonitis, and pleuritis. It also necessitates the placement of a postoperative chest tube, which contradicts the minimally invasive nature of OLIF [19,20]. Our findings are in line with these outcomes, further supporting the viability of upper lumbar OLIF as a reliable fusion strategy.

### 4.1. Extrapleural Approach

To overcome these drawbacks, the extrapleural lateral approach was developed and popularized by McCormick [21]. The author reported that the retropleural approach allows for the exposure of lesions from T3 to L1 without complications. Building on this, Uribe et al. demonstrated that the minimally invasive lateral retropleural thoracolumbar approach enables tumor resection and corpectomy while potentially reducing the complication rate compared with the traditional transpleural and endoscopic approaches [22]. Several recent studies have reported the use of a minimally invasive extrapleural lateral approach for OLIF in the upper lumbar spine. Mitsui et al. analyzed the predictors of the need for rib resection in OLIF at the upper lumbar level (L1–2 and/or L2–3) [6]. They suggested that rib resection should be considered when performing OLIF at L1–2. Furthermore, they reported an increased likelihood of rib resection in patients with thoracolumbar kyphosis (T10-L2 angle > 15.9°) or when the apex vertebra of the major coronal curve was located above L2. This is consistent with our findings, where rib resection was required in 53.3% of L1–2 procedures, whereas 83.3% of L2–3 procedures did not require rib resection. Several studies have reported on the performance of OLIF in the upper lumbar spine without rib resection. Tanasansomboon et al. reported a technical note demonstrating that single-level or sequential multilevel lateral interbody fusion at L1–2 can be performed without rib resection using intercostal subdiaphragmatic retroperitoneal access, with no reported complications [10]. Additionally, Lee et al. compared OLIF in the upper lumbar spine using the intercostal retroperitoneal approach without rib resection with conventional OLIF [9]. They reported that the intercostal retroperitoneal approach reduced the risk of endplate injury. However, this study did not specify whether rib resection was performed in the conventional OLIF group and lacked a comparison of radiological outcomes, except for endplate injury, as well as approach-related complications.

### 4.2. OLIF at Upper Lumbar Level

The present study is the first to compare upper- and lower-lumbar-level OLIF. There were no significant differences in demographic characteristics between the two groups. Notably, the operation time and EBL also showed no significant differences, suggesting that OLIF at the upper lumbar level can be performed in a minimally invasive manner. In the upper-level group, chest tube insertion was required in 11 cases (17.5%); however, no postoperative pulmonary complications were observed. Segmental artery injury (2.1%) and pseudo-hernia (4.2%) occurred at the L2–3 level; however, these are known complications of OLIF, and the incidence rates are consistent with those reported in previous studies [23,24]. These findings suggest that L1–2 and L2–3 levels may exhibit distinct anatomical and surgical characteristics, particularly in terms of approach-related morbidity. In our cohort, rib resection was more frequently required at L1–2, while pleural injuries and pseudo-hernia were observed exclusively at L2–3. These observations indicate that the upper lumbar spine should not be regarded as a single uniform entity and highlight the need for further studies to delineate level-specific complication patterns and refine surgical strategies for OLIF at each level.

The greater postoperative increase in ADH observed in the lower-level group may be attributable to the more frequent use of taller cages at these levels, as demonstrated by our analysis of cage height distribution (*p* < 0.001). Additionally, the relatively wider disc space and more accessible surgical corridor at L3–5 may have allowed for greater intraoperative distraction during cage insertion.

Importantly, the comparable fusion rates observed between the upper and lower lumbar groups suggest that OLIF can achieve similarly successful interbody fusion regardless of level, provided that appropriate surgical techniques and patient selection are ensured. Previous studies have reported that fusion rates in OLIF procedures generally exceed 85–90%, depending on cage type, bone graft material, and endplate preparation [25,26]. Similarly, no significant difference in cage subsidence was found between groups, despite the anatomical differences in vertebral body size and bone quality along the lumbar spine. This indicates that with skilled surgical techniques, subsidence can be effectively minimized even in anatomically constrained upper levels. In particular, achieving sufficient intraoperative disc space distraction is critical in reducing the risk of subsidence. This requires thorough disc removal, contralateral annular release, meticulous endplate preparation, and sequential trialing. Our results reinforce the importance of meticulous techniques over level-specific anatomy in achieving stable radiologic outcomes following OLIF [27].

### 4.3. Study Limitations

This study has several limitations. First, the retrospective nature of this study introduced a degree of uncertainty due to missing and erroneous data in the medical records, as well as a lack of clinical information. Moreover, variations in intervention techniques, such as direct or indirect decompression, open or percutaneous pedicle screw fixation, and the selection of cage sizes and angles, were not controlled. Second, the relatively small sample size, particularly, the limited number of L1–2 cases, may have limited the ability to detect small but clinically meaningful differences. Third, while descriptive changes in radiologic parameters, such as the disc angle and disc height, were observed within each group, our study design did not include a statistical analysis of pre- and postoperative changes. Incorporating within-group longitudinal assessments may have provided a more complete understanding of radiologic progression following OLIF at different levels. Fourth, this study did not analyze long-term clinical outcomes. While patient-reported outcomes and long-term functional recovery would have enriched the analysis, our study focused primarily on radiological outcomes and approach-related complications. Further prospective, large-population studies are needed to determine whether approach-related events affect clinical outcomes.

## 5. Conclusions

This study assessed approach-related complications in upper lumbar OLIF (L1–2 or L2–3) and compared its radiological outcomes with those of lower lumbar OLIF (L3–4 or L4–5). Although the extrapleural approach in OLIF at the upper lumbar level is often limited, the radiological outcomes were comparable to those of OLIF at the lower lumbar level. Clinically, this study suggests that OLIF is a safe and effective surgical option even for the upper lumbar spine and further supports its potential as a minimally invasive approach in patients requiring multilevel spinal fusion.

## Figures and Tables

**Table 1:** Demographic data.

	Upper Level (*n* = 63)	Lower Level (*n* = 60)	*p*
Age (years)	66.3 ± 11.9	65.9 ± 9.4	0.524
Sex (male:female)	24:39	22:38	0.509
Body mass index (kg/m^2^)	23.7 ± 3.3	24.1 ± 3.2	0.368
Bone mineral density (T score)	−1.23 ± 0.89	−1.12 ± 0.91	0.725
Smoking history (%)	13 (20.6)	14 (23.3)	0.443
Follow-up period (months)	59.6 ± 19.2	62.8 ± 17.0	0.695
Diagnosis (%)			0.486
Spondylolisthesis	29 (46.0)	23 (38.3)	
Spinal stenosis	21 (33.3)	19 (31.7)	
Degenerative disc disease	13 (20.6)	18 (30.0)	
Surgery level (%)			0.122
Single level	15 (23.8)	23 (38.3)	
Two level	48 (76.2)	37 (61.7)	
Operation time (min)	69.2 ± 21.2	58.1 ± 25.1	0.101
Estimated blood loss (mL)	95.6 ± 42.1	101.6 ± 49.2	0.451

Note: results are given as the number or the mean ± standard deviation.

**Table 2:** Approach-related events in upper OLIF.

	L1–2 (*n* = 15)	L2–3 (*n* = 48)
Rib resection (%)		
Not needed	7 (46.7)	40 (83.3)
11th	6 (40.0)	5 (10.4)
12th	2 (13.3)	3 (6.3)
Chest tube insertion (%)		
Intraoperative	3 (20.0)	6 (12.5)
Postoperative	1 (6.7)	1 (2.1)
Segmental artery injury (%)	0 (0)	1 (2.1)
Pseudo-hernia (%)	0 (0)	2 (4.2)

Note: results are given as the number or the mean ± standard deviation.

**Table 3:** Radiological parameters between upper and lower OLIF.

	Upper Level (*n* = 63)	Lower Level (*n* = 60)	*p*
ADH (mm)			
Preoperative	7.4 ± 2.3	7.6 ± 2.8	0.742
Postoperative 1 yr	11.7 ± 2.3	13.1 ± 3.2	**0.031**
PDH (mm)			
Preoperative	4.7 ± 2.6	4.9 ± 3.1	0.632
Postoperative 1 yr	5.7 ± 3.7	5.4 ± 2.8	0.586
Coronal disc angle (°)			
Preoperative	4.4 ± 4.5	2.8 ± 2.9	0.159
Postoperative 1 yr	2.4 ± 2.5	3.1 ± 2.9	0.241
Sagittal disc angle (°)			
Preoperative	7.4 ± 6.5	7.2 ± 4.5	0.871
Postoperative 1 yr	11.2 ± 5.2	11.4 ± 4.8	0.176
Cage parameters (%)			
Height			**<0.001**
8 mm	5 (7.9)	0 (0.0)	
10 mm	13 (20.6)	3 (5.0)	
12 mm	43 (68.3)	45 (75.0)	
14 mm	2 (3.2)	12 (20.0)	
Lordosis			0.285
6°	16 (25.4)	14 (23.3)	
12°	47 (74.6)	44 (73.3)	
18°	0 (0)	2 (3.3)	
Anterior position (mm)	4.9 ± 3.7	5.7 ± 3.8	0.120
Subsidence (%)	10 (15.9)	10 (16.7)	0.867
Fusion (%)	61 (96.8)	57 (95.0)	0.146

**Bold**: statistically significant. Note: results are given as the number or the mean ± standard deviation. ADH: anterior disc height; PDH: posterior disc height.

## Data Availability

Data are unavailable due to privacy or ethical restrictions.

## Abbreviations

The following abbreviations are used in this manuscript:

OLIF	Oblique lateral interbody fusion
ADH	Anterior disc height
PDH	Posterior disc height
